# CYP3A5基因多态性对异基因造血干细胞移植患者他克莫司血药浓度和不良事件的影响

**DOI:** 10.3760/cma.j.issn.0253-2727.2021.10.006

**Published:** 2021-10

**Authors:** 欣 陈, 荣莉 张, 卫华 翟, 巧玲 马, 爱明 庞, 栋林 杨, 祎 何, 嘉璘 魏, 尔烈 姜, 四洲 冯, 明哲 韩

**Affiliations:** 中国医学科学院血液病医院(中国医学科学院血液学研究所)，实验血液学国家重点实验室，国家血液系统疾病临床医学研究中心，天津 300020 State Key Laboratory of Experimental Hematology, National Clinical Research Center for Blood Diseases, Institute of Hematology & Blood Diseases Hospital, Chinese Academy of Medical Sciences & Peking Union Medical College, Tianjin 300020, China

**Keywords:** CYP3A5基因型, 他克莫司, 血药浓度, 造血干细胞移植, CYP3A5 genotype, Tacrolimus, Blood drug concentration, Hematopoietic stem cell transplantation

## Abstract

**目的:**

探讨在异基因造血干细胞移植（allo-HSCT）患者中，CYP3A5基因多态性与他克莫司血药浓度及急性移植物抗宿主病（GVHD）间的关系。

**方法:**

回顾性分析2019年7月至2020年2月在中国医学科学院血液病医院接受allo-HSCT的35例中国成人患者。移植前采集骨髓进行CYP3A5基因分型。应用静脉输注他克莫司、短疗程甲氨蝶呤（MTX）±吗替麦考酚酯进行GVHD预防。在他克莫司用药第2天或第3天监测初始血药浓度，随后每周监测2～3次。根据目标血药浓度（10～15 ng/ml）调整药物剂量。

**结果:**

16例携带CYP3A5*3/*3基因的allo-HSCT患者的初始他克莫司血药浓度（9.82 ng/ml对8.53 ng/ml）、初始血药浓度/剂量（C/D）比值（5.72 ng·ml^−1^·mg^−1^对4.26 ng·ml^−1^·mg^−1^）、allo-HSCT后第一和第二周C/D比值中位数（5.29 ng·ml^−1^·mg^−1^对4.61 ng·ml^−1^·mg^−1^，5.65 ng·ml^−1^·mg^−1^对4.56 ng·ml^−1^·mg^−1^）均明显高于19例至少携带一个CYP3A5*1等位基因的患者（*P*值分别为0.028、0.001、0.037、0.045）。至少携带一个CYP3A5*1等位基因的患者，allo-HSCT后Ⅲ～Ⅳ级急性GVHD的发生率有高于携带CYP3A5*3/*3基因患者的趋势［（26.3±10.1）％对（6.2±6.1）％，*P*＝0.187］。

**结论:**

CYP3A5基因型导向给药可能有助于allo-HSCT后更快地达到他克莫司目标血药浓度，减少严重急性GVHD的发生，改善移植疗效。

## Abstract (en)

**Objective:**

To investigates the relationship between CYP3A5 gene polymorphism, tacrolimus concentration, and acute graft versus host disease (GVHD) in patients undergoing allogeneic hematopoietic stem cell transplantation (allo-HSCT).

**Methods:**

A retrospective analysis of the clinical data of 35 Chinese adult patients who received allo-HSCT from July 2019 to February 2020 was conducted. Also, bone marrow samples were collected before transplantation for CYP3A5 genotyping, and intravenous infusion of tacrolimus and a short course of methotrexate (MTX) ± mycophenolate were used to prevent GVHD. The initial concentration was monitored on the second or third day of tacrolimus administration, followed by 2–3 times a week. The drug dose was adjusted according to the target blood concentration (10–15 ng/ml).

**Results:**

In 16 allo-HSCT patients with CYP3A5 *3/*3 gene, the initial concentration of tacrolimus (9.82 ng/ml *vs* 8.53 ng/ml), the initial concentration/dose (C/D) ratio (5.72 ng·ml^−1^ ·mg^−1^ *vs* 4.26 ng·ml^−1^·mg^−1^), and the median C/D ratio in the first two weeks after HSCT (5.29 ng·ml^−1^·mg^−1^ *vs* 4.61 ng·ml^−1^·mg^−1^, 5.65 ng·ml^−1^·mg^−1^ *vs* 4.56 ng·ml^−1^·mg^−1^) were significantly higher than in 19 patients with at least one CYP3A5 * 1 allele (*P*＝0.028, 0.001, 0.037, 0.045). The incidence of Ⅲ–Ⅳ aGVHD in patients with CYP3A5*1 alleles was higher than in patients with CYP3A5*3/*3 gene [(26.3±10.1)％ *vs* (6.2±6.1)％, *P*＝0.187].

**Conclusion:**

CYP3A5 genotype-directed administration may help achieve the target blood concentration of tacrolimus after HSCT more quickly, reduce the incidence of severe aGVHD, and improve the efficacy of transplantation.

异基因造血干细胞移植（allo-HSCT）是治疗多种血液系统疾病的有效方法。急性移植物抗宿主病（aGVHD）是allo-HSCT后危及生命的主要并发症之一，预防严重aGVHD对allo-HSCT的成功至关重要[1]。目前临床常以他克莫司联合甲氨蝶呤（MTX）作为allo-HSCT后预防移植物抗宿主病（GVHD）的标准方法之一[1]。但他克莫司的治疗窗很窄，考虑到药物疗效和毒性之间的平衡，临床实践中通常会进行血药浓度监测，但血药浓度监测存在滞后性，不适宜作为早期预测药物疗效的手段。

近年研究发现，药物代谢酶基因中的单核苷酸多态性（SNP）影响他克莫司的药代动力学。细胞色素P4503A4（CYP3A4）和CYP3A5参与他克莫司代谢的个体间差异。但在allo-HSCT患者中，CYP3A5基因多态性与他克莫司血药浓度间的关系的报道较少。因此，CYP3A5基因多态性对静脉输注他克莫司药代动力学的影响尚不清楚。本研究拟对CYP3A5的基因多态性和allo-HSCT后早期他克莫司静脉剂量、血药浓度、GVHD发生等方面进行综合分析，探讨能否通过检测CYP3A5基因多态性预测患者对药物的反应，避免药物不良反应。

## 病例与方法

1. 病例：回顾性分析2019年7月至2020年2月在我中心接受allo-HSCT的35例中国成人患者。其中男20例，女15例，中位年龄36（16～53）岁，急性髓系白血病（AML）23例，骨髓增生异常综合征（MDS）5例，急性B淋巴细胞白血病（B-ALL）2例，急性T淋巴细胞白血病（T-ALL）、肝脾T细胞淋巴瘤、皮肤T细胞淋巴瘤、混合表型急性白血病、再生障碍性贫血各1例。进行同胞全相合移植12例，单倍型移植21例，无关供者移植2例，均给予清髓预处理方案，后输注供者外周血造血干细胞。CYP3A4基因型均为TT。同胞全相合移植患者应用他克莫司（起始剂量0.03 mg/kg，从−1 d开始持续静脉输注）和短疗程MTX（+1 d 15 mg/m^2^静脉输注，+3和+6 d 10 mg/m^2^静脉输注）进行GVHD预防；单倍型及无关供者移植患者应用他克莫司（起始剂量0.03 mg/kg，从−5 d开始持续静脉输注）、短疗程MTX（+1 d 15 mg/m^2^静脉输注，+3、+6和+11 d 10 mg/m^2^静脉输注）和吗替麦考酚酯（MMF）（500 mg每12 h 1次，从−9 d开始持续静脉输注）进行GVHD预防。所有患者在预处理期间开始给予质子泵抑制剂静脉输注和预防真菌治疗。

2. 他克莫司药物浓度监测：应用酶放大免疫测定法在他克莫司用药第2天或第3天监测初始血药浓度，随后每周监测2～3次。根据目标血药浓度（10～15 ng/ml）调整药物剂量。

3. CYP3A5基因SNP位点分析：在allo-HSCT前获取患者骨髓标本，使用基因组DNA提取试剂盒［天根生化科技（北京）有限公司产品］提取患者的DNA，利用KAPA Hyper Prep Kit构建文库，采用Roche NimbleGen定制化试剂盒进行外显子目标区域捕获建库，之后用NovaSeq6000进行测序，下机数据与参考基因组比对之后通过变异检测软件GATK进行SNP相关位点分析。

4. 统计学处理：统计分析使用SPSS 22.0软件，采用Kaplan-Meier法进行生存分析，组间比较采用Log-rank检验。两组间各因素比较采用卡方检验和秩和检验。*P*<0.05为差异有统计学意义。

## 结果

1. 移植相关情况：35例患者移植后15例发生aGVHD，发生率42.9％，其中Ⅰ～Ⅱ度aGVHD 9例，Ⅲ～Ⅳ度aGVHD 6例。10例患者在应用他克莫司期间发生药物不良反应，主要为血糖、血压、肌酐（Cr）升高，肝功能异常及头痛。中位随访10.0（3.0～14.5）个月，31例生存，预期1年总生存（OS）率（88.6±5.4）％。共死亡4例，其中2例死于肺感染，1例死于脏器衰竭，1例死于复发。

2. CYP3A5基因多态性分布：35例患者中19例至少携带一个CYP3A5*1等位基因，16例携带CYP3A5*3/*3基因型。两组在年龄、性别、体重、诊断、供者类型、干细胞来源、人类白细胞抗原（HLA）差异、预处理方案、GVHD预防、联合抗真菌药物及移植前天冬氨酸转氨酶（AST）、丙氨酸转氨酶（ALT）、总胆红素（TBil）及Cr水平方面差异均无统计学意义（*P*值均>0.05）（表1）。

**表1:** 35例接受异基因造血干细胞移植患者的临床特征

特征	CYP3A5*1/*1或*1/*3（19例）	CYP3A5*3/*3（16例）	统计量	*P*值
年龄[岁，*M*（范围）]	34（16~53）	39（23~51）	1.591	0.112
性别（例，男/女）	12/7	8/8		0.506
体重[kg，*M*（范围）]	66.8（45.3~105.0）	60.5（48.8~92.0）	1.325	0.185
诊断（例）				0.901
AML/MDS	15	13		
ALL/淋巴瘤	3	2		
HAL	1	0		
AA	0	1		
供者类型（例）				1.000
MSD	7	5		
Haplo	11	10		
URD	1	1		
HLA相合点数（例）				0.971
10/10	8	6		
9/10	1	0		
7/10	1	1		
6/10	3	2		
5/10	6	7		
GVHD预防（例）				1.000
FK506	19	16		
MTX	19	16		
MMF	12	11		
真菌预防（例）				
第一周				1.000
卡泊芬净	18	15		
三唑类	1	1		
第二周				0.214
卡泊芬净	12	8		
三唑类	7	8		
第三周				1.000
卡泊芬净	8	7		
三唑类	11	9		
第四周				1.000
卡泊芬净	6	5		
三唑类	13	11		
ALT[U/L，*M*（范围）]	26.8（4.2~151.0）	151.2（8.2~331.0）	0.431	0.667
AST[U/L，*M*（范围）]	15.3（27.7~55.3）	18.5（7.8~189.1）	0.381	0.703
TBil[µmol/L，*M*（范围）]	（6.3~19.4）	10.5（5.9~34.5）	0.381	0.703
Cr[µmol/L，*M*（范围）]	48.1（28.6~77.0）	50.4（30.7~84.0）	0.050	0.960

注：AML：急性髓系白血病；MDS：骨髓增生异常综合征；ALL：急性淋巴细胞白血病；HAL：混合表型急性白血病；AA：再生障碍性贫血；MSD：同胞全相合移植；Haplo：单倍型移植；URD：无关供者移植；GVHD：移植物抗宿主病；FK506：他克莫司；MTX：甲氨蝶呤；MMF：吗替麦考酚酯；ALT：丙氨酸转氨酶；AST：天冬氨酸转氨酶；TBil：总胆红素；Cr：肌酐

3. CYP3A5基因多态性对allo-HSCT后初始他克莫司血药浓度和初始血药浓度/剂量（C/D）比值的影响：结果显示，携带CYP3A5*3/*3基因的患者初始他克莫司浓度及初始C/D比值明显高于携带CYP3A5*1等位基因的患者，差异均有统计学意义（*P*值均<0.05）（表2）。

**表2:** CYP3A5基因多态性对造血干细胞移植后初始他克莫司血药浓度和初始血药浓度/剂量（C/D）比值的影响［*M*（范围）］

组别	例数	初始他克莫司血药浓度（ng/ml）	初始C/D比值（ng·ml^−1^·mg ^−1^）
CYP3A5*1/*1或*1/*3	19	8.53（2.91~15.22）	4.26（1.79~5.89）
CYP3A5*3/*3	16	9.82（5.63~23.34）	5.72（3.31~9.01）

统计量		2.202	3.229
*P*值		0.028	0.001

4. CYP3A5基因多态性对allo-HSCT后前4周中位他克莫司C/D比值的影响：在allo-HSCT后的第1～2周，携带CYP3A5*3/*3基因的患者中位C/D比值均明显高于携带CYP3A5*1等位基因的患者（*P*值均<0.05）。然而，在allo-HSCT后3～4周期间，两组之间差异无统计学意义（*P*>0.05）（表3）。

**表3:** CYP3A5基因多态性对造血干细胞移植后前4周他克莫司血药浓度/剂量比值的影响［ng·ml^−1^·mg^−1^，*M*（范围）］

组别	例数	第1周	第2周	第3周	第4周
CYP3A5*1/*1或*1/*3	19	4.61（2.20～6.48）	4.56（2.18～7.63）	6.02（2.07～12.27）	7.74（3.02～13.16）
CYP3A5*3/*3	16	5.29（3.17～8.62）	5.65（4.76～11.02）	7.20（1.99～21.29）	7.98（3.22～36.12）

统计量		2.086	2.004	1.573	0.911
*P*值		0.037	0.045	0.116	0.362

5. allo-HSCT后CYP3A5基因多态性与aGVHD和肾功能损伤的关系：CYP3A5多态性倾向于成为影响allo-HSCT后严重（Ⅲ～Ⅳ度）aGVHD风险的因素之一，但不影响总体aGVHD发生率（表4，图1）。HSCT后共发生肾功能损伤3例，CYP3A5 *1/*1或*1/*3组发生1例，CYP3A5 *3/*3组发生2例，调整治疗后肾功能均获得改善。

**表4:** 造血干细胞移植后CYP3A5基因多态性与急性移植物抗宿主病（aGVHD）的关系（％，*x*±*s*）

组别	例数	aGVHD发生率	Ⅲ~Ⅳ度aGVHD发生率
CYP3A5*1/*1或*1/*3	19	47.4±11.5	26.3±10.1
CYP3A5*3/*3	16	37.5±12.1	6.2±6.1

*P*值 (Fisher确切概率检验)		0.734	0.187

**图1:**
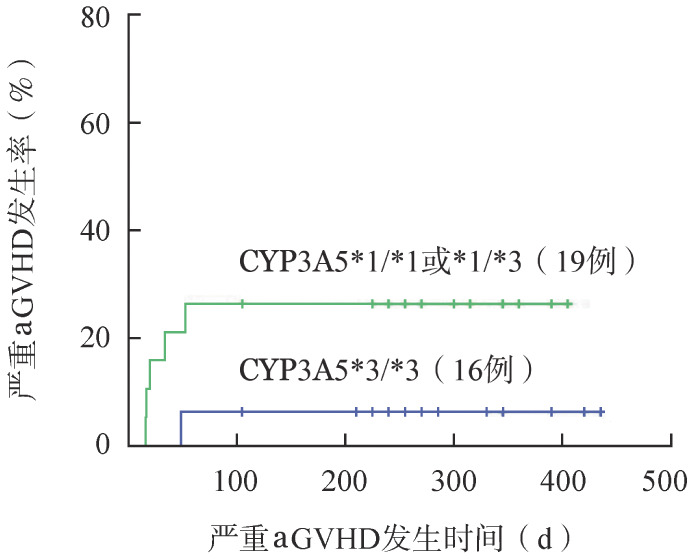
造血干细胞移植后CYP3A5基因多态性与严重急性移植物抗宿主病（aGVHD）的关系

## 讨论

allo-HSCT中用来预防GVHD的药物他克莫司，在推荐的初始静脉应用剂量为0.03 mg·kg^−1^·d^−1^的情况下，具有广泛的患者间药代动力学变异性[2]–[3]。血药浓度低于5 ng/ml预示着GVHD高风险，而血药浓度高于15 ng/ml增加他克莫司相关毒性如肾毒性和神经毒性等的发生[3]–[6]。特别是在allo-HSCT后的早期阶段，以期将他克莫司血药浓度稳定地维持在推荐的治疗窗口浓度5～15 ng/ml[7]。近年研究发现，药物代谢酶基因中的SNP影响他克莫司的药代动力学，在表达CYP3A5酶的个体中，CYP3A5可能在他克莫司的代谢中主导CYP3A4[8]–[12]。与功能变异相关的最重要的SNP是CYP3A5*3（6986A>G，rs776746），它会导致异常的mRNA剪接，从而产生非功能性的CYP3A5蛋白[13]–[14]。CYP3A5多态性频率的种族差异已被证实。据报道，CYP3A5*3/*3的频率在亚洲人中为65％～73％，在白种人中为87％～95％，在非裔美国人中为27％～50％[15]。既往大多数报道显示，在器官移植患者中CYP3A5*3基因型对他克莫司的药代动力学有显著影响[16]。此外，HSCT中常用的预防真菌药物，例如唑类抗真菌药物能够抑制CYP3A4酶[17]–[19]，产生药物之间的相互作用，且CYP3A5基因型的差异也可能会影响这些药物之间的相互作用。

CYP3A5*3（rs776746；6986A>G）是一种基因内SNP，它在内含子3中产生一个交替剪接的异构体，产生过早终止密码子，并产生无功能的CYP3A5酶，降低包括他克莫司在内的底物药物的代谢，导致药物血药浓度升高[20]–[23]。此前有报道显示这种SNP能独立影响他克莫司的药代动力学/药效学[23]–[25]。在肾、心脏和肺移植患者中，超过50项研究显示，与CYP3A5*3/*3基因型相比，携带CYP3A5*1/*1或CYP3A5*1/*3基因型的患者移植后他克莫司的血药浓度显著降低[23]。临床药理学实施联合会（CPIC）《实体器官移植患者指南》指出，携带至少一个CYP3A5*1等位基因的患者将获得较低的他克莫司稳态谷浓度，并延迟达到目标血药浓度[20]。在allo-HSCT患者中，他克莫司血药浓度与CYP3A5多态性相关研究的报道是有限的。Zhu等[26]在北卡罗来纳大学（UNC）骨髓移植（BMT）项目数据库共鉴定出295例成人allo-HSCT患者，纳入252例患者（包括白种人、黑种人）进行研究，结果显示，CYP3A5基因SNP与口服他克莫司平均稳态谷浓度之间存在显著相关性，与CYP3A5*3/*3患者相比，CYP3A5*1/*1患者需要更长时间达到他克莫司的目标血药浓度。Iwamoto等[27]进行了一项单中心前瞻性研究，选取2009年1月至2014年3月在密伊大学医院行HSCT并接受24 h持续静脉输注他克莫司的21例成人患者，观察HSCT后他克莫司C/D比值的变化与携带CYP3A5基因型的关系。结果显示，携带CYP3A5*1等位基因和CYP3A5*3/*3基因型的患者在HSCT后第14天（563 ng·ml^−1^·mg^−1^对742 ng·ml^−1^·mg^−1^，*P*<0.01）和第21天（672 ng·ml^−1^·mg^−1^对777 ng·ml^−1^·mg^−1^，*P*<0.05）的平均他克莫司C/D比值差异有统计学意义。我们的研究结果证实，中国（亚洲）人种HSCT后静脉他克莫司血药浓度与CYP3A5多态性的关系与之类似，16例携带CYP3A5*3/*3基因的患者初始他克莫司血药浓度、初始C/D比值、HSCT后第一和第二周中位C/D比值均明显高于19例至少携带一个CYP3A5*1等位基因的患者（*P*值分别为0.028、0.001、0.037、0.045）。但由于进行血药浓度监测并及时调整他克莫司剂量，CYP3A5基因多态性对HSCT后第三、四周C/D比值中位数无显著影响（*P*值分别为0.116、0.362）。

他克莫司已成为免疫抑制方案的基石，用于预防allo-HSCT后aGVHD的发生。研究表明，在植入前达到目标血药浓度是aGVHD的重要预测因子[28]–[29]。Khaled等[23]研究了以他克莫司/西罗莫司为基础预防GVHD的allo-HSCT患者，8例CYP3A5*1/*1基因携带者在allo-HSCT后100 d内Ⅱ～Ⅳ级aGVHD发生率明显高于CYP3A5*1/*3（40例）和CYP3A5*3/*3（122例）基因携带者（*P*＝0.04）。Yamashita等[30]报道了24例患者，在HSCT后100 d内，CYP3A5*1/*1或*1/*3基因型的患者（11例）Ⅲ～Ⅳ级aGVHD发生率也显著高于CYP3A5*3/*3（13例）基因型的患者（36％对0％，*P*＝0.017）。我们的研究结果显示，携带CYP3A5*1等位基因的患者，HSCT后Ⅲ～Ⅳ级aGVHD的发病率具有高于携带CYP3A5*3/*3基因的患者趋势（*P*＝0.187）。但两组患者总体aGVHD发生率差异无统计学意义（*P*＝0.734），这可能仍与定期根据血药浓度调整他克莫司用药剂量有关。我们的研究中，通过根据血药浓度调整他克莫司用药剂量，CYP3A5基因多态性对HSCT后第三、四周中位C/D比值已无明显影响，但即便如此，携带CYP3A5*3/*3基因患者仍然出现HSCT后aGVHD发生率降低的倾向，这种倾向可能与第一、二周中位C/D比值差异显著有关。与CYP3A5*3/*3患者相比，携带至少一个CYP3A5*1等位基因的患者他克莫司谷浓度显著降低，并且需要将他克莫司剂量提高至1.5～2倍才能达到相似的血药浓度水平[20]，因此在完善CYP3A5基因检测后，携带至少一个CYP3A5*1等位基因的患者，初始给药剂量可增加至1.5～2倍，以期早期、快速（HSCT后1～2周）达到稳定的目标他克莫司浓度，有利于减少HSCT后严重aGVHD的发生。

他克莫司诱导的肾毒性表现为急性肾损伤（AKI），是一种常见的治疗引起的毒性[31]。在Zhu等[26]的研究中，AKI与CYP3A4/5的任何SNP均无显著相关性。这种观察到的缺乏相关性可能归因于常规他克莫司血药浓度监测和日常肾功能评估，导致临床医师采取更积极的方法来减少AKI。我们的研究中，HSCT后未发生AKI，共发生肾功能损伤（Cr轻度升高）3例，CYP3A5 *1/*1或*1/*3组发生1例，CYP3A5 *3/*3组发生2例，调整治疗后肾功能均获得改善。

综上所述，CYP3A5基因型导向给药可使患者早期快速地达到目标血药浓度，减少严重aGVHD发生。而本研究的结果，也鼓励我们进一步针对中国（亚洲）人种开展HSCT后他克莫司血药浓度与CYP3A5多态性关系的多中心、前瞻性研究，以期验证不同种族中HSCT后他克莫司血药浓度与CYP3A5多态性的关系，指导HSCT患者临床用药，提高移植疗效。
